# The SUGAR handshake intervention to prevent hypoglycaemia in elderly people with type 2 diabetes: process evaluation within a pragmatic randomised controlled trial

**DOI:** 10.1186/s12877-025-06361-2

**Published:** 2025-10-01

**Authors:** Huda Y. Almomani, Helen M. Ayre, Richard A. Powell, Keivan Armani

**Affiliations:** 1 Isra University, Amman, Jordan; 2 University of Leeds, Leeds, UK; 3 Imperial College London, London, UK; 4 UCSI University, Kuala Lumpur, Malaysia

**Keywords:** Elderly, Type 2 Diabetes Mellitus, Hypoglycaemia, Randomised Controlled Trial, Intervention, Pharmacist, Process Evaluation

## Abstract

**Background:**

The SUGAR Handshake is a pharmacist-led educational intervention to prevent hypoglycaemia in elderly people with type 2 diabetes mellitus (T2DM). A process evaluation was conducted alongside the ROSE-ADAM pragmatic randomized controlled trial (RCT) to assess the implementation of the intervention and study procedures, explore mechanisms of impact, and examine future scalability.

**Methods:**

This mixed-methods process evaluation was nested within a single-centre RCT conducted at outpatient clinics in a Jordanian hospital. Routine monitoring quantitative data assessed adherence to the intervention components and study activities, and estimated reach. Qualitative data, collected through semi-structured interviews with 12 purposively selected participants on Days 45 and 90 of enrolment, captured experiences with the intervention and usual care. Thematic analysis was used for qualitative data; descriptive statistics and inferential tests were applied to quantitative data.

**Results:**

The intervention was well implemented: 104 of 106 participants (98.11%) continued the full intervention, with a 100% reach to those enrolled in the trial. Participants showed high adherence to study activities (mean ± SD: 88.07 ± 9.33 documented days on diaries; 77.97 ± 18.87 fasting blood glucose measurements). Intervention reach was 100%. Participants described the intervention as informative, easy to follow, and helpful in avoiding hypoglycaemia and the side-effects of antidiabetic medications. Key facilitators included trust in pharmacists, altruism, and social support. Reported barriers were people’s health status, age-related conditions, and stress.

**Conclusions:**

This process evaluation highlights the SUGAR Handshake’s potential for broader implementation and scale-up. By addressing identified barriers, future educational interventions may enhance adherence, improve patient outcomes, and advance hypoglycaemia management in diabetes care.

**Trial registration:**

Clinicaltrials.gov (NCT04081766), registration date 4,920,219.

**Supplementary Information:**

The online version contains supplementary material available at 10.1186/s12877-025-06361-2.

## Background

In 2021, approximately 529 million people were living with diabetes worldwide—a number projected to rise to 1.31 billion by 2050—making type 2 diabetes mellitus (T2DM) one of the most pressing global health challenges and a leading cause of death and disability globally [1]. Hypoglycaemia is a major adverse effect in people with diabetes that can lead to serious clinical, psychosocial and economic consequences [2, 3]. The risk of hypoglycaemia is highest in elderly people with T2DM, given several precipitating factors are associated with ageing (e.g., diminished counter-regulatory mechanisms, comorbidities, polypharmacy, impaired awareness of hypoglycaemia, cognitive impairment, and suppressed appetite) [4]. 

Educational interventions have been developed to control hypoglycaemia among people with diabetes that effectively reduce the severity and rate of hypoglycaemic attacks [5]. The lack of adequately powered clinical trials evaluating the effectiveness of hypoglycaemia educational interventions among people with T2DM [5], and the frequent exclusion of elderly people with diabetes from these trials [6], necessitate conducting high-quality pragmatic clinical trials (PCT) to assess the impact of education on this patient group.

Whilst RCTs are considered the gold standard of clinical research designs to generate evidence, they cannot provide information on the factors and processes influencing their outcomes. Process evaluation, however, allows the researcher to assess the implementation process of interventions and explore contextual factors and mechanisms influencing their functionality [9]. When developing and evaluating complex interventions, conducting process evaluations alongside clinical trials is recommended to improve the quality of the trial’s implementation and maximise interpretation of findings [9, 10]. 

Process evaluations have been conducted with several diabetes interventions, including those targeting prevention, self-management, and control of T2DM and its complications [11–13]. However, among RCTs assessing educational interventions for hypoglycaemia in people with T2DM, process evaluation measures are often limited to the quantitative evaluation of participants’ adherence to an intervention or diabetes knowledge [14–17]. Satisfaction questionnaires have also been employed [18, 19] but none of these studies conducted a comprehensive process evaluation encompassing multiple measures and used a mixed-methods approach. This study is, therefore, the first comprehensive investigation of participants’ adherence to an intervention and the study activities, and the processes that influenced the intervention’s effectiveness.

Details of the RCT protocol and its findings are published elsewhere [7, 20]. The aims and methods of the process evaluation were designed in accordance with the Medical Research Council’s (MRC) framework for complex interventions and process evaluation [9]. The process evaluation aimed to: (1) understand contextual factors influencing the implementation of the SUGAR Handshake intervention and RCT outcomes, (2) evaluate the implementation process and mechanisms underlying changes produced by the intervention, and (3) explore the future scalability of the RCT and the intervention.

## Methods

### Description of the ROSE-ADAM trial and SUGAR handshake intervention

A single-centre, pragmatic, open-label, randomised controlled trial (ROSE-ADAM RCT) was conducted in the outpatient clinics of a Jordanian hospital to evaluate the effectiveness of the SUGAR Handshake intervention in preventing hypoglycaemia among elderly individuals with T2DM [7]. Eligible participants were aged 65 years or older, diagnosed with T2DM, and receiving sulfonylurea, insulin, or at least three antidiabetic medications. They were randomly assigned in parallel to either the intervention group (SUGAR Handshake plus usual care) or a control group receiving usual care alone. The primary outcome was the total number of hypoglycaemic episodes per participant over a three-month follow-up period. Secondary outcomes included hypoglycaemia subtypes, incidence of any hypoglycaemia, hypoglycaemia-free survival, and fasting hyperglycaemia requiring therapy adjustment. Data were collected using self-monitoring glucose meters and structured hypoglycaemia diaries, and analysed according to the intention-to-treat principle. A total of 212 participants (mean age 68.98 years; 58.96% men) were randomly assigned equally to the two groups, with 89.6% completing the study.

The SUGAR Handshake intervention is a pharmacist-led, patient-centred, complex educational intervention designed to prevent hypoglycaemia by improving patients’ recognition, understanding, and self-management of hypoglycaemia-related risks. The intervention comprises five educational domains, represented by the acronym SUGAR: **S**igns and Symptoms, Understanding the Causes, Good Glycaemic Control, Acknowledgment, and Recap and Summary.

All domains were delivered through a one-on-one, face-to-face educational session, supported by a custom-designed pictogram tool to reinforce verbal communication and ensure clarity. The “Signs and Symptoms” domain covered early indicators of hypoglycaemia (e.g., shakiness, sweating, dizziness), using visual aids to help participants recognise and recall symptoms. The “Understanding the Causes” domain involved a tailored discussion of common individualised risk factors, such as missed meals, medication timing, overexertion, or illness, using scenario-based illustrations to personalise the content. The third domain, “Good Glycaemic Control and Self-Monitoring”, provided practical guidance on blood glucose monitoring practices, interpretation of results, and target ranges. Participants were instructed to measure fasting blood glucose (FBG) at least three times per week, and more frequently if hypoglycaemia was suspected. Instructions were provided on the proper use of glucose meters and completion of a structured diary to document blood glucose readings and symptoms. The “Acknowledgment” domain focused on fostering patient responsibility and engagement by encouraging participants to recognise their role in preventing hypoglycaemia and applying the strategies discussed. Finally, the “Recap and Summary” domain reinforced the key messages of all previous components and helped participants consolidate their learning through verbal review and a printed take-home summary. Participants in both the intervention and control groups received a follow-up phone call on Day 45 to remind them to continue measuring their FBG and documenting their readings and any symptoms in the provided diaries. For participants in the intervention group, these calls also served to reinforce the educational session content, revisit key self-care behaviours, and address any implementation challenges they faced.

The intervention was delivered by 2 clinical pharmacists with formal training in diabetes care and behaviour change communication. Prior to implementation, the pharmacists received structured training covering diabetes and hypoglycaemia education, use of the pictogram, patient counselling strategies, and motivational interviewing principles. The educational content and tools were piloted and refined during the development phase to ensure cultural relevance and acceptability for the target population.

A pragmatic approach guided both the trial and the intervention design, using the PRagmatic Explanatory Continuum Indicator Summary-2 (PRECIS-2) tool [8] to ensure the intervention was suitable for real-world clinical settings. The SUGAR Handshake was shown to reduce the rate and incidence of hypoglycaemia significantly and improve hypoglycaemia-free survival among elderly people with T2DM, without increasing the incidence of fasting hyperglycaemia. The CONSORT flow diagram and full methodology of the RCT and intervention details are available in the main trial publication [7].

### Study setting and design

This process evaluation was nested within the pragmatic ROSE-ADAM RCT [7]. A mixed-methods approach was adopted for this process evaluation, involving both qualitative—using participants’ interviews—and quantitative evaluation, using routine monitoring data. The Consolidated criteria for reporting qualitative research (COREQ) were applied while reporting the qualitative part of this study [21]. 

**Fig. 1:**
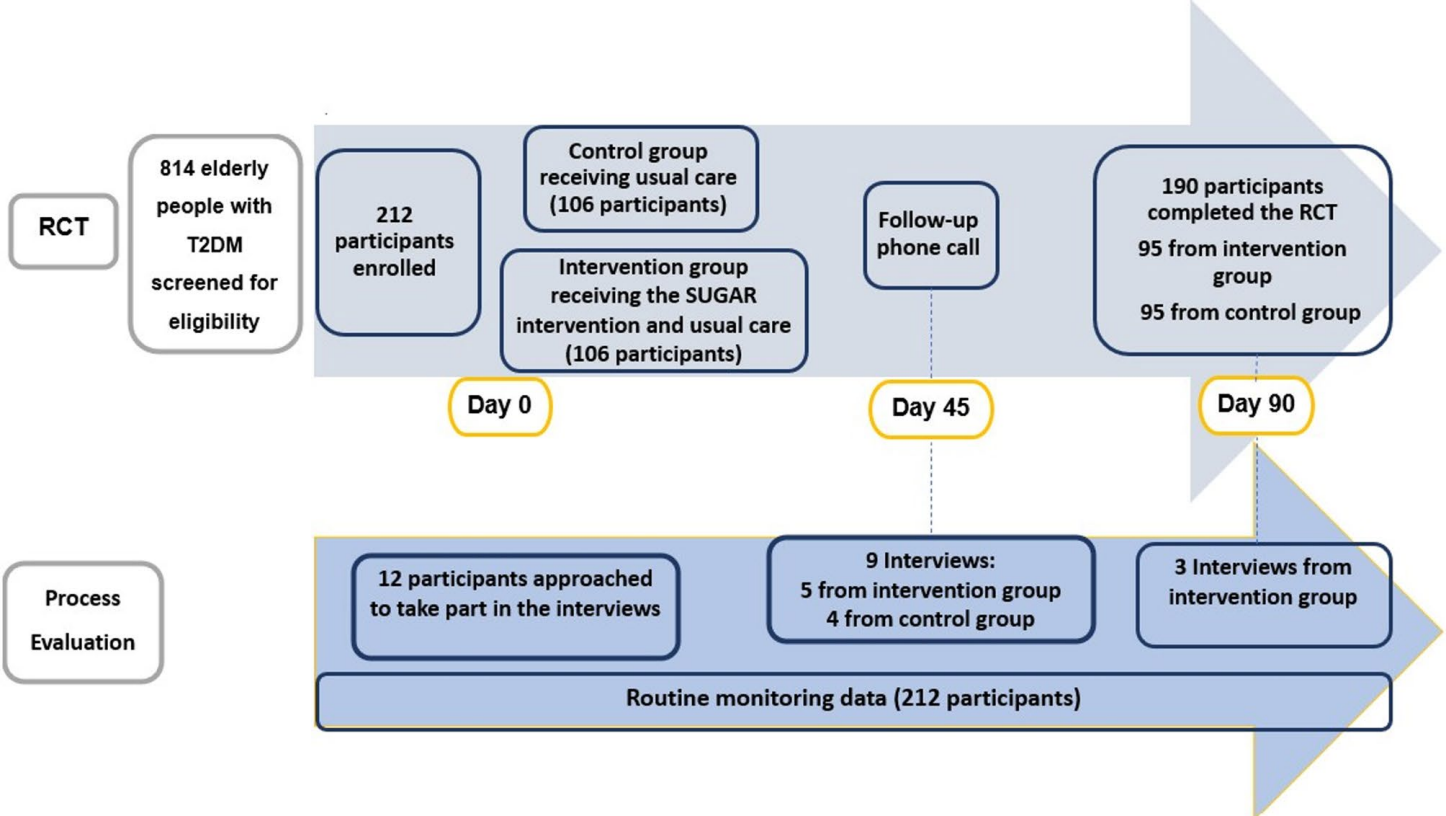
The RCT and process evaluation timeline

Eligible participants for the qualitative element were identified from participants who enrolled in the RCT. Purposive sampling was used on day 45 to select participants from both the intervention and control groups, including those who had and had not experienced hypoglycaemia, and to ensure representation across different outpatient clinics. This approach aimed to capture a range of experiences with the intervention and usual care. On day 90, purposive sampling focused on participants in the intervention group who experienced the highest number of hypoglycaemic episodes, in order to explore possible explanations for the frequency of events, including any discontinuation or inconsistent use of intervention components. This sampling approach aligns with process evaluation methodology, where the goal is to gain and in-depth understanding of variations in participant responses and intervention implementation (Fig. 1).

Semi-structured interviews were undertaken (HYA and AA) over the telephone. The quantitative evaluation complemented the qualitative approach by assessing the extent of participants’ adherence to the intervention and the study activities: i.e., FBG measurements and hypoglycaemia diaries throughout the study duration. Evaluation of participants’ adherence to the intervention served as an indicator of whether success or failure in implementing the intervention was the reason for the effectiveness/ineffectiveness of the intervention in preventing hypoglycaemia [9]. Reach refers to the extent to which the intervention components and study procedures were delivered to, and engaged with, by participants enrolled in the trial. It was measured as the proportion of trial participants who received the intervention (educational session and pictogram) and study materials (glucose meters, hypoglycaemia diaries, and follow-up phone calls). Participants’ engagement in these activities reflected the feasibility of both the intervention and the study. Data on eligible individuals who declined participation or were not approached were not collected; thus, assessing reach beyond the enrolled population falls outside the scope of this evaluation.

The interview process followed the theoretical saturation concept, starting with three initial interviews and then adding one interview at a time [22]. Interviews were continued until sufficient responses were collected to achieve a comprehensive understanding of the study objectives, the collected data represented the outlined themes in the analytical framework, and no new insights emerged from further data analysis [22, 23]. Median interview duration was 17 (range 15–20) minutes.

Out of the 212 participants enrolled in the RCT, a total of 12 participants were interviewed: nine on day 45 and three on day 90. All participants who were approached to take part in the interviews agreed to participate. Day 45 was chosen as the midpoint of the total duration of the RCT to allow the reinforcement of the intervention while permitting the intervention to be re-considered for modification, if needed. Of these, eight were from the intervention arm and four from the control arm. On average, participants were 66.63 years old (± 2.2). Ten participants were male and eleven were living in a multi-generational setting. In one interview, a participant was accompanied by his adult son, who occasionally contributed to the conversation to clarify or elaborate on the participant’s responses. While the son was not an independent interviewee and is not counted among the 12 participants interviewed, his input was retained where it added relevant contextual insight. Participants’ characteristics are presented in Table 1.

**Table 1:** Characteristics of interview participants

Characteristics	Intervention group, n=8	Control group, n=4
Clinic, n (%)		
Endocrinology	6 (75%)	2 (50%)
Cardiology	1 (12.5%)	1 (25%)
Diabetic foot care	1 (12.5%)	1 (25%)
Age (years), Mean ± SD	66.63 ± 2.2	66.63 ± 2.2
Sex, n (%)		
Male	7 (87.5%)	3 (75%)
Female	1 (12.5%)	1 (25%)
Educational level, n (%)		
Primary school	2 (25%)	0 (0%)
Secondary school	3 (37.5%)	1
University and higher	3 (37.5%)	3
Living arrangement n, (%)		
Alone	1 (12.5%)	0 (0%)
With only a partner	0 (0%)	0 (0%)
Multi-generational setting	7 (87.5%)	4 (100%)
Duration of diabetes (years), Mean ± SD	17.88 ± 8.20	15 ± 4.20
Self-monitoring of blood glucose, n (%)		
Yes	7 (87.5%)	4 (100%)
No	1 (12.5%)	0 0%)
Prior hypoglycaemia, n (%)		
Yes	5 (62.5%)	4 (100%)
No	3 (37.5%)	0 (0%)

*SD*standard deviation

### Interview guides and procedures

The interview guide was created by the research team (Supplementary Tables 1 and Table 2), matching the objectives of the process evaluation. Interviews were audiotaped, transcribed, then translated verbatim from Arabic to English by one researcher (HYA) fluent in both languages. Transcripts were translated back from English into Arabic by an independent professional translator to ensure their accuracy, with anomalies between both versions resolved through discussion. Transcripts were not returned to participants. Before data collection, the interview guide underwent both content and face validation. It was piloted with two experienced researchers in qualitative studies at Al-Balqa Applied University, Jordan, as well as two elderly people with T2DM who took part in the trial, to confirm the relevance and clarity of the interview questions.

### Data analysis

#### Qualitative analysis

Interview transcripts were analysed using a deductive approach. A working analytical framework [‘codebook’] was developed before starting the analysis process, guided by the MRC framework for complex interventions (Supplementary Table 3). The analytical framework consisted of four domains with respect to the process evaluation objectives and the theoretical frameworks used to design the SUGAR handshake. The analysis was conducted using a six-phase ‘codebook’ thematic analysis process [21] to apply and identify codes, categories and themes, using NVivo 12 software [24]. HYA conducted analysis and coding of all transcripts. Peer debriefing was undertaken by HA, leading to the refinement of the final themes through discussion and mutual consensus.

#### Quantitative analysis

Adherence to the intervention and study activities was measured by referring to hypoglycaemia diaries and FBG readings (Supplementary Table 4). Reach was evaluated by measuring the level of contact between the participants with the modes of delivery of the intervention and with study activities (Supplementary Table 4). Quantitative analysis of routine monitoring data was conducted using RStudio version 3.4.2 (28-9-2017). Comparisons between study groups were performed using Fisher’s Exact test and unpaired t-test. The conducted analyses were two-tailed, with a p-value of < 0.05 and a 95% confidence level.

### Trial registration

The trial was registered at Clinicaltrials.gov (NCT04081766), registration date: 4-9-20219.

## Results

**Fig. 2:**
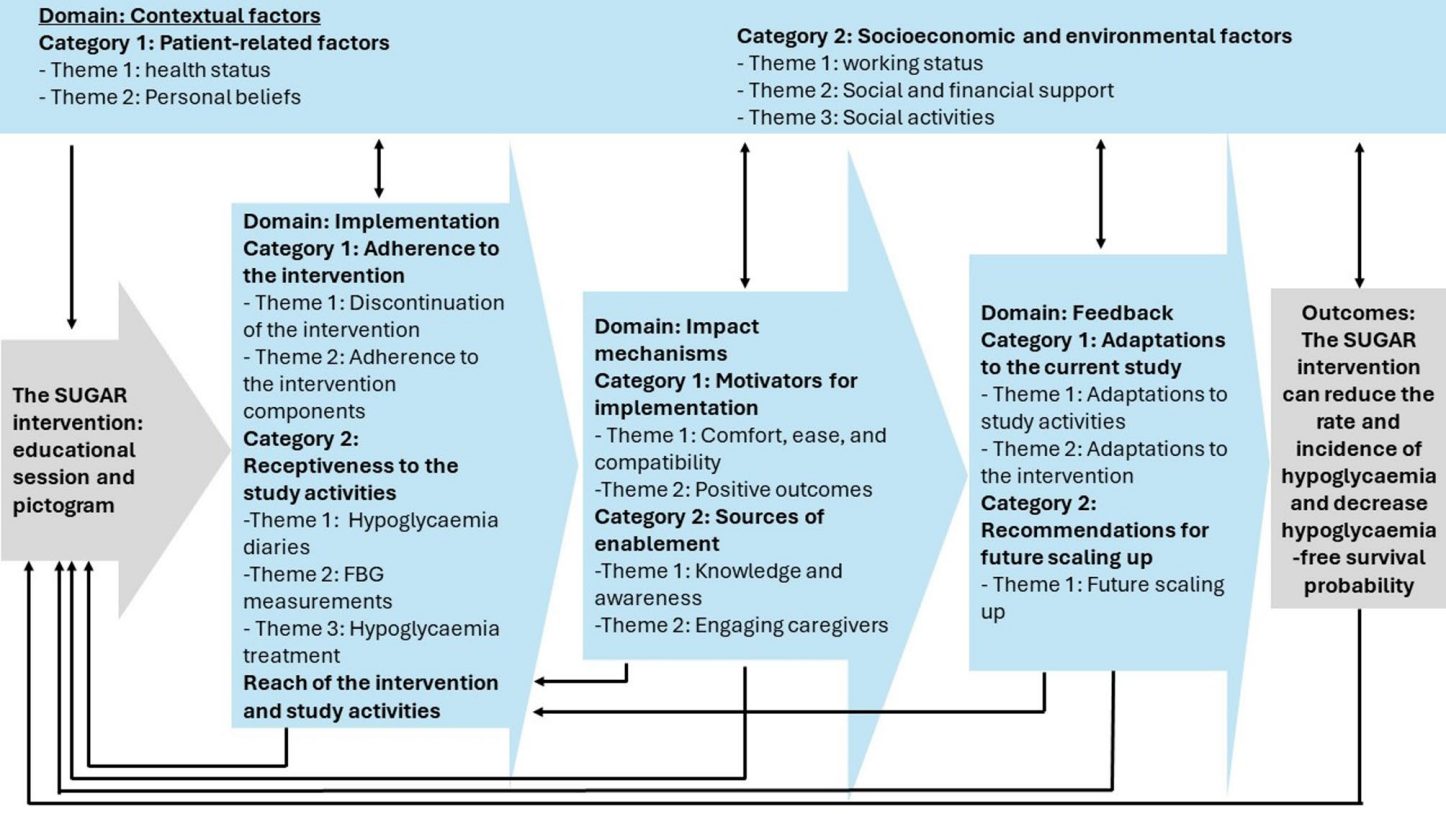
Conceptual framework of participants’ engagement with the SUGAR intervention, mapped to the MRC framework for process evaluation. The diagram illustrates four primary domains (contextual factors, implementation, impact mechanisms, and feedback), their categories and themes, and how they influence the intervention’s reach, adherence, and outcomes.

Findings suggest multiple factors influenced participants’ engagement with the intervention and study activities. Categories and themes were identified, as illustrated in Fig. 2, and presented in four primary thematic domains: contextual factors; impact mechanisms; feedback, and implementation.

### Domain 1: Contextual factors

This domain involved personal or external circumstances influencing participants’ commitment during the RCT and consisted of two categories: patient-related factors, and socioeconomic and environmental factors.

### Category 1: Patient-related factors

Patient factors were personal determinants influencing the RCT’s implementation and intervention and were identified as two themes: health status and personal beliefs.

### Theme 1: Health status

Health problems arising from ageing and comorbidities negatively affected the implementation of study activities and the intervention. Being ill or in pain acted as a barrier to frequent measuring of blood glucose (BG) levels.

*“In some days*,* my father is tired or in pain*,* so we don’t measure his blood glucose level”*. (son of 65–69/M, intervention group)

Additionally, ageing-related conditions (e.g., trembling hands) interfered with the use of test strips.

*“I have a shortage in the test strips because sometimes my hand shakes when I insert the test strip into the glucose meter. So*,* it doesn’t display a reading on the glucose meter screen”.* (65–69/M, intervention group).

Psychological health seemed to impact adherence to the intervention. One participant discontinued the intervention due to depressive feelings after a relative’s death. Also, eating preferences and appetite may interfere with adherence to the intervention’s dietary component.

### Theme 2: Personal beliefs

Altruism prompted participants to continue their engagement in the study and intervention. Some felt a connection to scientific research, which influenced their adherence, hoping their contribution would bring benefits through the knowledge produced.

*“I have felt it is necessary to follow the instructions for your sake as a researcher*,* so you can get the findings you are looking for”. (65–69/M*,* intervention group)*

### Category 2: Socioeconomic and environmental factors

Socioeconomic and environmental factors also influenced participants’ adherence and were grouped into three themes: working status, social and financial support, and social activities.

### Theme 1: Working status

Working status was noted to potentially impact adherence to study activities.

*“I remember that the study duration is three months*,* right? I believe it is a little bit long. No doubt it could be fine with many patients who have plenty of time*,* but for some*,* it may be considered long*,* especially if they work”. (65–69/M*,* control group)*

### Theme 2: Social and financial support

The presence or absence of a family caregiver influenced participants’ adherence to measuring BG and to using hypoglycaemia diaries.

*“Sometimes we miss measuring his blood glucose level because no one of us (adult children) is there with him to do so and my mother does not know how to use the glucose meter”. (Son of 65–69/M*,* intervention group)*

Some participants expressed their financial inability to buy test strips, which they considered expensive and prevented them from frequent BG monitoring.

### Theme 3: Social activities

Social activities are common among elderly Jordanians (including visits, occasions, and gatherings) at which sweets are served, influencing participants’ adherence to the intervention’s dietary component.

*“Following the instructions is completely OK with me because I am always at home. Especially nowadays*,* with this coronavirus outbreak*,* there is no fast food*,* restaurants*,* parties*,* or occasions. You know such things are common among peasants”*. (65–69/M, intervention group)

### Domain 2: Impact mechanisms

This domain encompassed effective processes underpinning adherence to the RCT and intervention and consisted of two categories: motivators for implementation and sources of enablement.

### Category 1: Motivators for implementation

Factors reinforcing participants’ performance in the study were classified in three themes: comfort, ease, and compatibility; positive outcomes; and the desire to avoid negative outcomes.

### Theme 1: Comfort, ease, and compatibility

Some participants found the study or intervention comforting, convenient, and compatible with their lifestyles, leading to positive emotions.

*“I feel measuring my blood glucose is psychologically comforting”. (65–69/M*,* intervention group)*

*“… I was comforted with the time of medications’ administration you have proposed to me”. (70–74/M*,* intervention group)*

### Theme 2: Positive outcomes

Some participants reported regular measurements helped them control their BG levels and consequently improved their glycaemic status and health. Achieving tangible pleasant outcomes and feeling better seemed to reinforce participants’ adherence.

*“I feel my health has improved not got worse… I am motivated to continue with the measurements”*. (65–69/M, control group)

### Theme 3: Desire to avoid negative outcomes

Some participants in the control group were concerned about deterioration in glycaemic status, either by experiencing hyper- or hypoglycaemia, which motivated them to keep self-monitoring their BG and confirm symptomatic hypoglycaemia.

*“When I was experiencing fatigue*,* sweat*,* and tremors*,* I didn’t pay attention to measuring my blood glucose level. However*,* I have become compliant to measuring my blood glucose level on daily basis now and I have become able to detect low blood glucose levels”*. (70–74/F, control group)

### Category 2: Sources of enablement

Mediators influencing participants’ psychological and physical abilities to perform study-related activities were grouped into two themes: knowledge and awareness; engaging caregivers.

### Theme 1: Knowledge and awareness

Some participants reported improvement in their ability to recognise and confirm hypoglycaemia through low BG levels or symptoms after taking part in the study:

“*I have become compliant to measuring my blood glucose level on a daily basis now and I have become able to detect low blood glucose levels…*.” (70–74/F, control group).

### Theme 2: Engaging caregivers

Some family caregivers took responsibility for administering the intervention and study-related activities by helping participating family members.

“*We have started measuring his blood glucose in the morning and at night and things have been ok…*.” (son of 65–69/M, intervention group).

### Domain 3: Feedback

This domain related to participants’ suggested changes to increase the intervention’s effectiveness and acceptability of the study activities. This domain consisted of two categories: adaptations to the current study and recommendations for future scaling-up.

### Category 1: Adaptations to the current study

Adaptations undertaken in the study were identified in two themes: adaptations to study activities and adaptations to the intervention.

### Theme 1: Adaptations to the study activities

One participant considered daily BG monitoring difficult to follow:

“*Frankly*,* I have found it hard to measure my blood glucose every day. This is a high frequency for me*,* so I have started to measure it two to three times a week*.” (70–74/M, intervention).

Although participants were encouraged to monitor their FBG level daily—particularly when hypoglycaemia was suspected—the study protocol instructed them to do so at least three times per week. This recommendation aimed to balance the need for sufficient data collection with participant burden and support adherence to BG monitoring. During the enrolment visit, some participants expressed concerns about the feasibility of daily monitoring. In response, the study approach was adapted to emphasize flexibility, guiding participants to monitor their FBG at a minimum of three times per week while allowing more frequent monitoring based on individual capacity or need.

### Theme 2: Adaptations to the intervention

A treatment-related problem that involved taking two medications from the same group of anti-diabetic medications was identified during a telephone call on day 45 for one participant, which was causing frequent hypoglycaemic attacks. An adaptation was made for this participant’s intervention content and the participant was contacted by telephone at day 90 to re-evaluate hypoglycaemia and the amended intervention.

### Category 2: Recommendations for future scaling-up

Adaptations to scale up the intervention and the study activities in the future were represented in one theme: Future scaling-up.

### Theme: Future scaling-up

Some participants wished to be contacted more frequently (e.g., on a weekly or monthly basis). Participants reported that frequent follow-up times are necessary for better glycaemic monitoring and more enhanced intervention recommendations.

“*You also told me at the inclusion visit that you are going to contact us after a month or more*,* so why wouldn’t you amend the frequency to be every two weeks?*” (son of 65–69/M, intervention group).

“*There should be a follow-up for the patient. It is not wrong if you call the patient every week*.” (70–74/M, intervention group).

### Domain 4: Implementation

This domain involved participants’ adherence to the intervention and the study activities. Participants’ adherence was classified into two categories: adherence to the SUGAR Handshake, and receptiveness to study activities. The implementation domain also involved quantitative evaluation of the reach of the intervention and study activities.

### Category 1: Adherence to the SUGAR handshake intervention

Adherence to the intervention was represented in two themes: discontinuation of the intervention and adherence to the intervention components.

### Theme 1: Discontinuation of the intervention

Most participants in the intervention group (104/106, 98.11%) adhered to the intervention. One participant discontinued the intervention as a result of experiencing depressive feelings from the loss of a relative, while another discontinued because she felt better.

### Theme 2: Adherence to the intervention components

Participants expressed different levels of adherence to recommendations concerning their anti-diabetic medications. Some participants reported compliance with the time needed to take their medications during the day and regarding meals.

“*Before participating in your study*,* I was taking my anti-diabetic medications irregularly. In sometimes before meals and in other times after meals. But after you educated me*,* I have become compliant to the times of taking my medications*”. (70–74/M, intervention group)

### Category 2: Receptiveness to study activities

Participants’ engagement in the study activities was represented in three themes: hypoglycaemia diaries, FBG measurements, and hypoglycaemia treatment.

### Theme 1: Hypoglycaemia diaries

Routine monitoring data revealed the majority of participants (91.04%) returned their diaries, with comparable numbers in the intervention and control groups (97 vs. 96).

Participants who returned their diaries (*n* = 193) were highly adherent to using their diaries (Supplementary Table 5). On average, participants documented 88.07 ± 9.33 days of their participation duration, with no statistically significant difference between the study groups (*p* = 0.771).

### Theme 2: FBG measurements

Participants presented high levels of adherence to measuring FBG levels (Supplementary Tables 6 and Table 7). One-third of participants (33.16%) fully adhered to FBG measurements, while the majority (92.23%) adhered to at least 40% of FBG measurements during the study. The adherence measures to FBG levels did not significantly differ between the intervention and control groups. Some participants reported they developed a habitual routine of monitoring their FBG levels, which reinforced their adherence:

“*During my whole life*,* I was not measuring my blood glucose every day. However*,* now I am measuring it every day. When I wake up*,* I directly insert the test strip into the glucose meter and measure my blood glucose*.“(65–69/M, intervention group).

### Theme 3: Hypoglycaemia treatment

Some participants stated they adhered to the corrective actions of hypoglycaemia whenever they experienced low BG levels or hypoglycaemia symptoms.

“*When I was feeling the blood glucose level is low before I go to sleep*,* I was having a glass of juice or an apple*,* then the blood glucose becomes normal in the next morning… I didn’t experience dangerous symptoms or sweating but when my blood glucose level is 120 or 90. (*70–74/M, intervention group)

### Reach of intervention and study activities

The reach measure—referred to the proportion of participants who received the educational session and the pictogram from participants in the intervention group, and the proportion of participants who received the glucose meters and hypoglycaemia diaries from participants in both groups—was 100% (Supplementary Table 8). The reach to participants through the telephone calls was also high, with most participants answering the day 45 (95.28% intervention group, 96.04% control group) and day 90 (96.23% intervention group, 95.96% in control group) calls.

## Discussion

This nested process evaluation within the ROSE-ADAM RCT [7] aimed to explore participants’ engagement in the SUGAR Handshake intervention and the scalability of this PCT. While previous clinical trials evaluating the effect of educational interventions on preventing hypoglycaemia in people with diabetes involved quantitative evaluation of only one aspect (e.g., participants’ adherence to the interventions’ components [14–17] or participants’ satisfaction) [18, 19], this is the first process evaluation systemically guided by the MRC framework for evaluating educational intervention on hypoglycaemia prevention in elderly people with T2DM.

The process evaluation demonstrated that educational sessions and pictograms were effectively delivered to the intervention group, with a 100% reach, maximising the effectiveness of the intervention [9]. Participants showed high levels of adherence to the intervention components, especially the recommendations regarding anti-diabetic medications and diet.

The SUGAR Handshake intervention was integrated into participants’ daily routines. Consistent with previous studies, the intervention helped participants develop habitual behaviours through repetition, contributing to sustained adherence [25, 26]. Participants found the instructions of the intervention easy to follow. There is strong evidence that health recommendations in an accessible language improve participants’ engagement in self-care and medication management [27, 28]. The intervention also improved participants’ knowledge and awareness of medication management, motivating adherence to anti-diabetic medications’ recommendations. The relationship between better diabetes knowledge and enhanced performance of diabetes self-care activities is also confirmed by previous studies [29, 30]. The desire to avoid hypoglycaemia and improve insulin injection skills also motivated adherence to the intervention similar to previous comparable studies [31, 32]. Participants were highly adherent to FBG monitoring and documenting on the hypoglycaemia diaries, indicating the feasibility of using these methods in detecting and documenting hypoglycaemic attacks. The instructions concerning hypoglycaemia diagnosis and treatment, and FBG monitoring, improved participants’ knowledge and motivated them to continue FBG monitoring. Participants in both groups had similar adherence levels to measuring FBG and the hypoglycaemia diaries, which reduced the risk of bias in reporting hypoglycaemia in the RCT [7]. 

This evaluation identified several contextual factors that worked as facilitators or barriers to implementing the intervention and the study activities. Altruism, consistent with previous research in people with diabetes [33, 34], served as a facilitator, promoting participants’ engagement. Participants’ trust in pharmacists promoted adherence, consistent with studies highlighting patients’ confidence in healthcare professionals providing education and medication management [35–37]. Social support from family caregivers facilitated practising of the intervention and the study activities. This is supported by two systematic reviews showing that engaging family members in behavioural interventions improves glycaemic control, medication adherence, and knowledge among people with T2DM [38, 39]. Clinical and healthcare interventions should consider the involvement of family members in the education and training of elderly patients to achieve diabetes control and minimise complications.

The evaluation revealed that worsened health status and age-related conditions were barriers to adherence. This could be explained by the impact of such conditions on limiting the physical or psychological capability to perform diabetes self-care tasks, such as BG monitoring [40, 41]. Furthermore, stressful life events and depressive feelings impaired adherence to the intervention. Previous research links these findings with disturbing the patients’ psychological health, appetite disruption, and causing pain and fatigue, which reflect non-adherence to medications, diet, physical activity, and the self-monitoring of BG [42–45]. 

Reduced appetite and food preferences limited some participants’ adherence to the intervention’s dietary recommendations. Decline in appetite amongst elderly people could be age-related, including loss of hunger, alteration in taste, difficulty in chewing, and malabsorption [46, 47]. Accordingly, more individualising of the dietary and anti-diabetic medication recommendations is necessary among patients with limited appetite to foster the intervention’s effectiveness.

### Considerations for scaling-up

The scale-up strategy of the intervention is developed with attention to maintaining those elements contributing to the intervention’s effectiveness, including the core components (anti-diabetic medications and dietary recommendations), intervention characteristics (individualisation, ease, and compatibility), and methods of delivery (educational session, pictograms, and follow-up phone calls) while minimising barriers. Recommendations to improve participants’ adherence to the intervention and maximise its effectiveness as concluded from the process evaluation, are detailed in Table 2. To scale up the SUGAR Handshake intervention, stakeholders should focus on creating an appropriate framework for the intervention to be consistently and adequately delivered by pharmacists across different healthcare settings (Table 3).

**Table 2:** Recommendations to improve the intervention effectiveness

Domain		Challenges	Recommendations
Medications-related	• Changes in anti-diabetic medications type or regimen. • Treatment-related problems (inappropriate medication, doubling therapy, drug-drug interactions, medications causing hyperglycaemia)		• More frequent follow-up through phone calls to detect changes and treatment-related problems and to amend the intervention accordingly • Collaboration with physicians to amend prescribed medications
Dietary-related	• Limited appetite and food preferences		• Encourage the use of high carbohydrate-containing food in these patients^a^. • Collaborate with physicians to modify anti-diabetic medications based on patients’ appetite and food preferences
Health status	• Psychological distress • Limited cognitive ability or functionality to perform the intervention.		• Consider more engagement of family caregivers to motivate patients’ adherence, or to implement the intervention. • More frequent follow-ups to motivate adherence.
Financial support	• Expensive test strips		• Consider educating patients on prioritising BG testing during critical times such as in the morning, before meals, or at bedtime rather than regular monitoring
Social life	• Patients’ engagement in social occasions and activities		• Consider educating patients on nutrition management, by focusing on carbohydrate counting, portion control, and meal planning strategies. • Offer suggestions for diabetic-friendly menu options before the social events • Consider reminders for time of medications’ administration • More motivation techniques to adhere to dietary instructions

^a^[48]; *DDI* drug-drug interaction

**Table 3:** Recommendations for the stakeholders to scale up the intervention

Challenges	Stakeholders	Recommendations
Hypoglycaemia-specific patient education	Healthcare sectors and settings	Integrate the SUGAR Handshake intervention into routine diabetes care protocols.
Effective educational skills and knowledge to deliver the intervention	Schools of Pharmacy, Jordan Pharmacists Association, healthcare sectors	Provide comprehensive training and education programs for pharmacists to ensure consistent delivery of the intervention across different healthcare settings.
Quality improvement of the intervention	Healthcare sectors	Establish mechanisms for ongoing evaluation and quality improvement to assess the scalability, effectiveness, and sustainability of the intervention in different healthcare settings.
Financial constraints	Healthcare sectors	- Provide information on available resources for individuals facing financial constraints, such as local organisations that offer support for diabetes management supplies - Explore options for generic or lower-cost alternatives to branded test strips.

Before a nationwide roll-out of our findings can be undertaken, further research is needed that is not possible with the current study. Specifically, first, a cost-effectiveness study should compare the direct expenses of pharmacists’ time, pictograms and glucose strips with the savings expected from fewer hypoglycaemic events; second, an equity analysis is necessary to stratify adherence and outcomes by socioeconomic status, sex and rural-versus-urban clinic to determine whether the intervention reduces or exacerbates disparities in diabetes care; third, external validation through a multi-site pilot in routine primary-care settings—implemented by regular staff without researcher support—is necessary to test whether the high fidelity observed in the trial is sustainable under real-world conditions. Addressing these three areas would provide policymakers with rigorous evidence on cost, equity and generalisability that can support informed decision-making to scale-up the intervention.

### Strengths and limitations

A main strength of this process evaluation was the adoption of the MRC framework, which provided a solid foundation for guiding the implementation and a systematic and comprehensive evaluation of the SUGAR Handshake intervention. The evaluation offered insight into the effectiveness of the intervention and provided valuable insights for potential replication or scaling-up in future research. Additionally, analysing the process evaluation findings before analysing the RCT outcome helped minimise bias in the interpretation of outcomes, ensuring a more objective and robust evaluation.

This study has several limitations. First, the process evaluation relied solely on qualitative data to assess participants’ implementation of the intervention. While this provided valuable in-depth insights, the absence of a quantitative measure of adherence to intervention components limits the ability to assess implementation fidelity objectively. Future research should consider integrating quantitative methods alongside qualitative approaches to evaluate adherence more comprehensively. Second, there is the possibility of a social desirability response bias, as participants might have provided answers they perceived as more socially acceptable. However, efforts were made to minimise this bias by creating a non-judgmental and accepting environment during the interviews.

Third, the small sample size of interviewees may have constrained the diversity of perspectives captured. However, the integration of qualitative insights with the broader quantitative trial data provided useful context for interpreting participants’ experiences and helped contextualise the process evaluation findings. Lastly, the qualitative sample was not fully representative of the trial population in terms of gender and household type.

## Conclusion

The findings of this process evaluation indicate that the SUGAR Handshake intervention for elderly people with diabetes was well delivered and received and could be scaled up and implemented more widely. Ultimately, the evaluation is a stepping stone for further research and advancements in hypoglycaemia management interventions. By addressing the identified barriers and leveraging the strengths of this evaluation, future educational interventions can be designed to promote adherence and improve patient outcomes, ultimately contributing to the enhancement of diabetes care and the overall well-being of individuals with T2DM.

## Supplementary Information

Supplementary Material 1.

## Data Availability

The datasets used and/or analysed during the current study are available from the corresponding author on reasonable request.

## Declarations

### Corresponding author

Correspondence to Keivan Armani.
