# Supplementary material for: The SUGAR handshake intervention to prevent hypoglycaemia in elderly people with type 2 diabetes: process evaluation within a pragmatic randomised controlled trial
Source: BMC Geriatr. 2025 Oct 1;25:753. doi: 10.1186/s12877-025-06361-2 (PMC12486974; doi:10.1186/s12877-025-06361-2)
Supplement: Supplementary file 1 — Supplementary Material 1. [file 12877_2025_6361_MOESM1_ESM.docx]

**Supplementary Table 1: Interview guide for participants in the intervention group**

| 1. **Introduction**   You have previously read in the participant information sheet that we are conducting a phone interview as a part of this study and you accepted to participate in it. Therefore, I would like to ask you some questions about your experience with the study processes and the intervention components that have been provided to you at the educational session. The information will help us in improving several aspects of the study and the intervention. The interview should take about 10 minutes. Are you available to respond to the questions at this time? |
| --- |
| 1. I would like to start by asking: how do you describe your participation in the study and the intervention so far? If needed, the interviewers may explain the question by the follow-up question: How do you rate your participation in the study and the intervention from good to poor and why? |
| 1. What has worked for you from the study processes and the intervention components that you were asked to do?   – why do you think they have?  What hasn't worked for you from the study processes and the intervention components that you were asked to do?  - why do you think they haven't? |
| 1. From your perspective, how the study and the intervention could be improved? Please consider any aspects of the study that you think could be improved. |
| 1. At the end, I would like to thank you for taking part in this interview. If you have any questions or concerns, please contact me on my mobile number provided in the participant information sheet. |

**Supplementary Table 2: Interview guide for participants in the control group**

| 1. **Introduction**   You have previously read in the participant information sheet that we are conducting a phone interview as a part of this study and you accepted to participate in it. Therefore, I would like to ask you some questions about your experience with the study processes that have been provided to you at the inclusion visit. The information will help us in improving several aspects of the study. The interview should take about 10 minutes. Are you available to respond to the questions at this time? |
| --- |
| 1. I would like to start by asking: how do you describe your participation in the study so far? If needed, the interviewers may explain the question by the follow-up question: How do you rate your participation in the study from good to poor and why? |
| 1. What has worked for you from the study processes that you were asked to do?   – why do you think they have?  What hasn't worked for you from the study processes that you were asked to do?  - why do you think they haven't? |
| 1. From your perspective, how the study could be improved? Please consider any aspects of the study that you think could be improved. |
| 1. At the end, I would like to thank you for taking part in this interview. If you have any questions or concerns, please contact me on my mobile number provided in the participant information sheet. |

**Supplementary Table 3: Generated framework using the deductive approach for the process evaluation**

| **Framework Domains** | **Categories** |
| --- | --- |
| **Contextual factors** | Patient-related factors  Socioeconomic and environmental factors |
| **Mechanisms of impact** | Motivators for implementation  Sources of enablement |
| **Feedback** | Adaptations to the current study  Recommendations for future scaling-up |
| **Implementation** | Adherence to the SUGAR handshake intervention components  Receptiveness to the study activities  Reach of the SUGAR handshake intervention and study activities |

**Supplementary Table 4: *Quantitative evaluation measures of the process evaluation***

| Quantitative measures | | Indicators of the quantitative measures |
| --- | --- | --- |
| Adherence by participants | Adherence to the intervention | Proportion of participants who adhered to the intervention at day 45 and day 90. |
|  | Adherence to the study activities | **Hypoglycaemia diaries**  - Number of documented days in 90 days.  - Proportion of participants with full adherence in 90 days  - Proportion of participants with at least 80% adherence (at least 72 days)  **Blood glucose measurements**  - Number of FBG measurements for the first follow-up, second follow-up, and total follow-up duration^a^.  - Proportion of participants with full adherence in the first, second, and total follow-up duration^a^.  - Proportion of participants with at least 80% adherence^b^.  - Proportion of participants with at least 40% adherence^c^. |
| Reach | Reach to the intervention | - Proportion of participants who received the educational session and pictogram out of these randomised to the intervention group. |
|  | Reach to the study activities | - Proportion of participants who received glucose meters, hypoglycaemia diaries, and phone calls |
| ^a^ first follow-up: 1-45 days, second follow-up: 46-90 days, total follow-up: 1-90 days, ^b^at least 80% adherence :36 days in the first and second follow-up, 72 days in the total follow-up, ^c^ at least 40% adherence :19 days in the first and second follow-up, 39 days in the total follow-up, FBG: fasting blood glucose | | |

**Supplementary Table 5: Adherence levels to hypoglycaemia diaries**

| Measures of adherence | Intervention group (n=97) | Control group (n=96) | p  value | Total (n=193) |
| --- | --- | --- | --- | --- |
| Number of documented days, Mean ± SD | 88.27± 8.17 | 87.88 ±10.42 | 0.771 | 88.07±9.33 |
| Full adherence (90 days), n(%) | 89 (91.75) | 87 (90.63) | 0.805 | 176 (91.19) |
| At least 80% adherence (=>72 days), n(%) | 94 (96.91) | 94 (97.92) | 1.000 | 188 (97.41) |
| n= sample size, SD: standard deviation | | | | |

**Supplementary Table 6: Adherence to fasting blood glucose measurements across the follow-up points**

| Measures of adherence | Follow-up 1  (1-45 days) | | | | Follow-up 2  (46-90 days) | | | |
| --- | --- | --- | --- | --- | --- | --- | --- | --- |
|  | IG  (n=97) | CG  (n=96) | p  value | Total  (n=193) | IG  (n=97) | CG  (n=96) | p value | Total  (n=193) |
| Number of FBG measurements, Mean ± SD | 41.77±7.36 | 40.82±8.35 | 0.403 | 41.30±7.86 | 35.93±13.14 | 37.45±12.17 | 0.406 | 36.68  ±12.66 |
| 100% adherence, n(%) | 61  (62.89) | 50  (52.08) | 0.147 | 111  (57.5) | 39  (40.21) | 35  (36.46) | 0.658 | 74  (38.43) |
| At least 80% adherence, n(%) | 86  (88.66) | 81  (84.38) | 0.407 | 167  (86.53) | 68  (70.10) | 73  (76.04) | 0.418 | 141  (73.06) |
| At least 40% adherence, n(%) | 94  (96.91) | 92  (95.83) | 0.721 | 186  (96.37) | 83  (85.57) | 86  (89.58) | 0.514 | 169  (87.56) |
| Complete non-adherence, n (%) | 0 (0.00) | 0 (0.00) | NA | 0 (0.00) | 4 (4.12) | 4 (4.17) | 1.000 | 8 (4.15) |
| CG: control group, IG: intervention group, n: sample size, NA: not available, SD: standard deviation | | | | | | | | |

**Supplementary Table 7: Adherence to fasting blood glucose measurements across the total study duration**

| Measures of adherence | Total follow-up (1-90 days) | | | |
| --- | --- | --- | --- | --- |
|  | IG (n=97) | CG (n=96) | p-value | Total (n=193) |
| Number of FBS measurements, Mean ± SD | 77.67±18.38 | 78.27±19.44 | 0.826 | 77.97  ±18.87 |
| 100% adherence, n(%) | 33 (34.02) | 31 (32.29) | 0.879 | 64 (33.16) |
| At least 80% adherence, n(%) | 76 (78.35) | 77 (80.21) | 0.859 | 153 (79.27) |
| At least 40% adherence, n(%) | 90 (92.78) | 88 (91.67) | 0.795 | 178 (92.23) |
| Complete non-adherence, n(%) | 0 (0.00) | 0 (0.00) | NA | 0 (0.00) |
| CG: control group, IG: intervention group, n: sample size, NA: not available, SD: standard deviation | | | | |

**Supplementary Table 8: The reach measures of the intervention and study activities**

| Reach measures | Intervention group, n/n (%) | | Control group, n/n (%) |
| --- | --- | --- | --- |
| The intervention |  | |  |
| Educational session at baseline  Pictogram at baseline | | 106 /106 (100)  106/106 (100) |  |
| The study activities | | | |
| Glucose meters  hypoglycaemia diaries | | 212/212 (100)  212/212 (100) |  |
| Phone call at day 45 | | 101/106 (95.28) | 102/106 (96.23) |
| Phone call at day 90 | | 97/101 (96.04) | 95/99 (95.96) |
| n: sample size | |  |  |
